# Fatal Encephalitis Caused by Cristoli Virus, an Emerging Orthobunyavirus, France

**DOI:** 10.3201/eid2606.191431

**Published:** 2020-06

**Authors:** Christophe Rodriguez, Guillaume Gricourt, Melissa Ndebi, Vanessa Demontant, Lila Poiteau, Sonia Burrel, David Boutolleau, Paul-Louis Woerther, Vincent Calvez, Sebastian Stroer, Jean-Michel Pawlotsky

**Affiliations:** Henri Mondor Hospital, Assistance Publique des Hôpitaux de Paris at University of Paris-Est, Créteil, France (C. Rodriguez, G. Gricourt, M. Ndebi, V. Demontant, L. Poiteau, P.-L. Woerther, J.-M. Pawlotsky);; Pitié-Salpêtrière Hospital, Assistance Publique des Hôpitaux de Paris at Sorbonne-Université, Paris, France (S. Burrel, D. Boutolleau, V. Calvez, S. Stroer)

**Keywords:** orthobunyavirus, shotgun metagenomics, meningitis/encephalitis, viruses, Cristoli virus, France

## Abstract

We report the discovery of a new orthobunyavirus, Cristoli virus, by means of shotgun metagenomics. The virus was identified in an immunodepressed patient with fatal encephalitis. Full-length genome sequencing revealed high-level expression of a virulence factor, possibly explaining the severity of the infection. The patient’s recent history suggests circulation in France.

The *Orthobunyavirus* genus of the *Peribunyaviridae* family contains numerous viruses, usually transmitted by mosquitoes (*1*). New members are regularly discovered through mosquito screening campaigns, but most of them are not pathogenic (*2*–*4*). Global warming implies changes in the distribution of their vectors, possibly exposing humans to the onset of new diseases.

Broad-spectrum diagnostic tests, such as metagenomics methods, are useful to discover new viruses, especially in patients with encephalitis of unknown etiology (*2*,*5*). We have developed an original method based on shotgun metagenomics (MetaMIC) for the diagnosis of bacterial, viral, fungal, and parasitic infections from any human fluid or tissue (*6*). We used it to discover a previously unknown member of the *Orthobunyavirus* genus of the *Peribunyaviridae* family, Cristoli virus, in a patient with fatal encephalitis. The study was approved by the Créteil Institutional Review Board (Créteil, France).

## The Study

A 58-year-old woman living in the Paris, France, area was hospitalized in September 2018 for isolated fever resistant to amoxicillin/clavulanic acid. She had a history of complete remission of non-Hodgkin lymphoma, decompensated cirrhosis related to autoimmune hepatitis treated with sirolimus, possibly congenital hypogammaglobulinemia associated with B-lymphopenia, and episodes of lower respiratory tract and digestive infections in the years preceding her admission. She last traveled to Italy and on a Mediterranean cruise in summer 2017. Although her fever declined on ofloxacin, she was admitted again in October 2018 for deterioration of her general health, anorexia, and psychomotor retardation. Her neurologic symptoms worsened during the following 6 months. Multiple electroencephalograms showed nonspecific slowing of background activity. Magnetic resonance imaging revealed limbic and cerebellar abnormalities compatible with inflammatory or infectious encephalitis (Figure). Results of testing for multiple cerebrospinal fluid samples showed only an increase in α interferon; no microbial agent was identified. A cerebral biopsy was performed in March 2019 for shotgun metagenomics testing. The patient’s condition continued to deteriorate, and she died in the intensive care unit on March 27, 2019.

**Figure:**
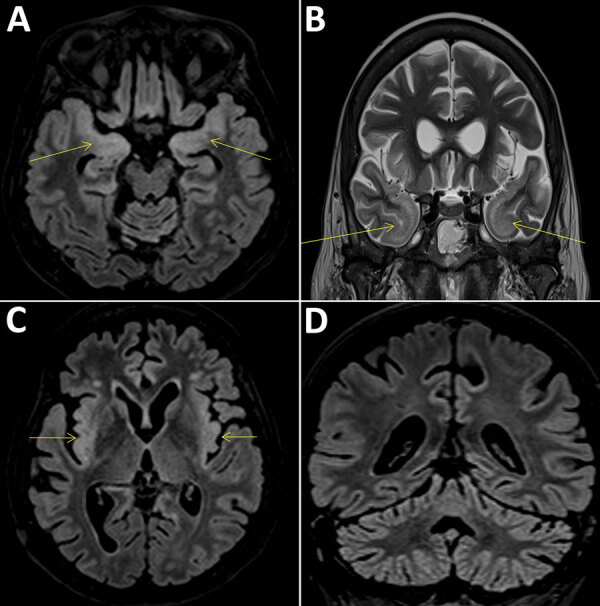
Cerebral magnetic resonance imaging scans compatible with the diagnosis of encephalitis in a 58-year-old woman, France. Fluid-attenuated inversion recovery (FLAIR) and T2 hypersignals in limbic system structures, including both amygdalae (A, arrows), temporal poles (B, arrows), and insular cortex (C), associated with FLAIR hyperintensities of the cerebellar cortex (D).

We ground the cerebral biopsy material in a sterile isotonic solution. We performed preextraction using bead beating combined with chemical cell disruption, then a combined DNA/RNA extraction using QIAsymphony (QIAGEN, https://www.qiagen.com). We tested a negative environmental control (isotonic sterile solution) in parallel. We prepared DNA libraries with Nextera XT DNA, and RNA libraries with RNA Human RiboZero TruSeq Stranded Total RNA Library Prep Kit (Illumina, https://www.illumina.com). We pair-end sequenced libraries, 2 × 150 bp, with High Output Kit version 2 on a NextSeq500 device (Illumina). We analyzed the sequences with MetaMIC software (*6*). 

Identification of nonhuman sequences uses different databases, including cleaned National Center for Biotechnology Information nucleotide and nonredundant databases (GenBank release 229, December 2018) that contain all known microorganisms. For new species, we conducted verification using an algorithm combining de novo assembly with Meta-SPAdes version 3.12.0 (http://cab.spbu.ru/software/meta-spades) and contig alignment with the ViPR database (https://www.viprbrc.org). After manual curation of full-length viral sequences, we performed alignment and phylogenetic analyses with the closest known viral species using Muscle version 3.8.31 (http://www.drive5.com/muscle) and IQ-Tree version 1.3.11.1 (http://www.iqtree.org). We used the general time reversible plus gamma 4 plus invariate sites model of nucleotide substitution and 10,000 full maximum-likelihood bootstrap replicates.

Shotgun metagenomics revealed the presence of sequences from a new member of the *Orthobunyavirus* genus of the *Peribunyaviridae* family; the sequences were not present in the negative environmental control. This new virus was named Cristoli virus after the city of Créteil where it was described. We reconstructed the full-length sequence of the viral genome de novo in silico. The large (L), medium (M), and small (S) segments were individualized and could be aligned with similar sequences from known orthobunyaviruses (*1*) (Appendix 1 Figure 1, panels A, B). Cristoli virus segregated within serogroup Turlock; the closest related virus was Umbre virus. These viruses cluster on a different branch from all other known orthobunyaviruses with a 100% bootstrap (Appendix 1 Figure 1, panel C). Cristoli virus differed from Umbre virus by 11.4% of L, 12.3% of M, and 7.0% of S segments (differences with other orthobunyaviruses) (Appendix 2).

We identified a 6,783-bp open reading frame (ORF), coding for a protein of 2,261 aa in the L segment, which, by analogy with other orthobunyaviruses, corresponded to the RNA-dependent RNA polymerase (Appendix 1 Figure 1). The conserved H….PD….DxK orthobunyavirus catalytic domain (*7*) was present between positions 37 and 101, identical to that of Umbre virus. Umbre and Cristoli viruses differed by 44 aa in the RNA polymerase (Table).

**Table:** Amino acid differences coded by open reading frames in Cristoli virus compared with Umbre virus*

Open reading frame			
Large	Medium	Small	Nonstructural
A16T, M135I, H138Y, Q231L, S249A, R252K, E252D, P318H, V382I, N384T, E408D, V425I, K433R, A452T, R477K, G488E, V566I, V568I, N630S, I792V, V904I, E931S, T939A, R942K, T943S, I966V, V1080I, R1206K, I1444V, G1481D, K1566R, K1662R, D1665H, N1669D, G1987S, AQ2058V, D2074E, N2085D, K2130R, T2168I, T2232S, L2255F, T2258I, G2270E,	M2V, V3I, S10L, A12V, L14F, S16N, R32K, D99N, V203I, I332V, A338I, C340Y, V358I, I376V, V382I, K390R, N393S, G427E, R451K, M452L, A458V, I465L, D468N, A473T, A474T, V484I, D504N, K511R, V514E, V515I, S521G, M546V, S578N, T594I, T627A, M692V, N701D, S724L, T746V, P758del, R759del, T760P, K761R, I762V, R772K, S773L, I790V, G791D, V827I, D837E, V839I, S847N, N853D, I860V, S888K, A889T, A904M, V921I, T923M, A967S, V989A, V994I, N996S, I1002M, I1008V, V1020M, T1031A, N1168D, F1206Y, T1254A, T1322M, S1323T, T1378I, T1406A, R1419K, T1443A, A1444T, N1446S	F12Y A53P T87A S115N	H3R L52P R56K

*Differences are indicated using the formula X#Y, where X indicates the presence of an amino acid in Umbre virus, Y a different amino acid in Cristoli virus, and # the position of the difference. A, alanine; C, cysteine; D, aspartic acid; E, glutamic acid; F, phenylalanine; G, glycine; H, histidine; I, isoleucine; K, lysine; L, leucine; M, methionine; N, asparagine; P, proline; Q, glutamine; R, arginine; S, serine; T, threonine; V, vazline; Y, tyrosine.

In segment M, we identified a 4,336-bp ORF coding for a protein of 1,445 aa, corresponding to a polyprotein processed into the 2 envelope glycoproteins n and c (Gn and Gc) and nonstructural protein m (NSm) in orthobunyaviruses (Appendix 1 Figure 1). Cristoli and Umbre viruses differed by 78 aa in the M segment, including 2 deletions (P758del and R759del) (Table). R759del corresponds to a trypsin cleavage site used for bunyavirus characterization (*8*). The conserved RxxR cleavage motif at the C-terminus of the Gn domain was conserved in Cristoli virus at positions 298–301. All conserved cysteine positions (*9*) were also conserved.

We identified 2 overlapping ORFs in the S segment, one corresponding to the nucleocapsid (N) protein (714 bp, 238 aa), the other to nonstructural protein s (NSs, 240 bp, 80 aa). We found few differences with Umbre virus for these proteins (Table).

The amounts of viral RNAs we semiquantified by shotgun metagenomics were 5.4 log viral genomes/mg of biopsy for segment L, log viral genomes/mg of biopsy 5.0 for segment M, and 6.2 log viral genomes/mg of biopsy for segment S. For confirmation, we designed specific reverse transcription PCRs for Cristoli virus segments L, M, and S. Brain extracts were positive for all three, with amplicon sequences identical to those in the full-length genome (Appendix 1 Figure 2). We deposited the annotated genome sequence into Genbank (accession nos. MN488996 [S segment], MN488997 [M segment], and MN488998 [L segment]).

## Conclusions

The etiologic diagnosis of encephalitis is difficult because >25% of cases remain of unknown cause (*5*). Diagnostic methods without prior knowledge of the microorganisms sought, such as metagenomics, detect infectious agents when other techniques have failed (*10*). We used an original shotgun metagenomics approach and in-house software MetaMIC to discover a new virus belonging to the *Orthobunyavirus* genus of the *Peribunyaviridae* family in an immunodepressed patient with slowly progressive fatal encephalitis. Cristoli virus is close to Umbre virus, a member of the Turlock serogroup not previously associated with human disease. Because the patient had not traveled during the year preceding the onset of her symptoms, our findings suggest that Cristoli virus is endemic to France. Like other members of its family, it could have been transmitted through a mosquito bite. Changes in the distribution of mosquito species related to climate change may make such transmissions more frequent in the future. In this context, the metagenomics method we developed is particularly useful to detect unusual or emerging infectious agents in patients with unassigned infectious syndromes.

Full-length sequence analysis of Cristoli virus revealed a classical *Peribunyaviridae* genome organization with conserved motifs. The genome segment coding for the NSs protein was found in an amount 10 times higher than that of the other segments. Because NSs has been described as a virulence factor (*11*,*12*), its high-level expression in a context of immune depression could explain the unusual severity of the disease. If efficacious antiviral treatments become available (*13*), a rapid etiologic diagnosis of orthobunyavirus infections will become necessary. The shotgun metagenomics method we developed fulfills the criteria for such routine diagnosis.

Appendix 1 Additional methods and figures for fatal encephalitis caused by Cristoli virus, France.

Appendix 2 Additional information about fatal encephalitis caused by Cristoli virus, France.
