# Supplementary material for: Fatal Encephalitis Caused by Cristoli Virus, an Emerging Orthobunyavirus, France
Source: Emerg Infect Dis. 2020 Jun;26(6):1287–90. doi: 10.3201/eid2606.191431 (PMC7258463; doi:10.3201/eid2606.191431)
Supplement: Appendix 1 — Additional methods and figures for fatal encephalitis caused by Cristoli virus, France. [file 19-1431-Techapp-s1.pdf]

Article DOI: https://doi.org/10.3201/eid2606.191431

Fatal Encephalitis Caused by Cristoli Virus, an Emerging Orthobunyavirus, France

Appendix 1

Supplemental Materials and Results

Specific RT-PCR Method for Amplification of Segments S, M, and L and Population Sequencing of the Amplicons

Based on the viral sequences obtained by CMg, we designed an RT-PCR method for each of the 3 segments to confirm the presence of the bunyavirus in the brain biopsy sample. RT-PCR were carried out from 5uL of brain biopsy extract using 3 pairs of primers for segments S, M and L (SegS.F: AGCTGCGATGGCTAAACTCA and SegS.R: GCAATGTAGCCAGAAAGCCG; SegM.F: TGGGTTCAGCAATCAAGCGA and SegM.R: CAGACGCAGTGTATCCCACA; SegL.F: TCACGAACTGCCTACTGCAA and SegL.R: CTTCTTGTCGAGCCGCATTG) and the One-Step RT-PCR kit (QIAGEN, https://www.qiagen.com) following the manufacturer's instructions. We reverse-transcribed and amplified each mix using one program for segments S and M (50°C, 60 min; 95°C, 15 min; {95°C, 30 s; 56°C, 30 s; 72°C, 1min} × 40; 72°C, 10min) and another program for segment L (50°C, 60 min; 95°C, 15 min; {95°C, 30 s; 55°C, 30 s; 72°C, 1 min} × 40; 72°C, 10 min] on a Veriti thermocycler (ThermoFisher, https://www.thermofisher.com). We used a negative control for each RT-PCR. PCR products were purified with NucleoFast kit (Macherey-Nagel, https://www.mn-net.com) and sequenced using BigDye Terminator version 3.1 Cycle Sequencing Kit (ThermoFisher) on ABI3130 (ThermoFisher).

Segment-Specific RT-PCR and Population Sequencing Results

Migration of PCR products on an agarose gel showed the presence of 3 amplicons of 150 bp corresponding to segment S, 780 bp corresponding to segment M, and 857 bp corresponding to segment L (Appendix 1 Figure 2). Population sequencing of the PCR products showed full identity with the viral sequence generated by shotgun metagenomics.

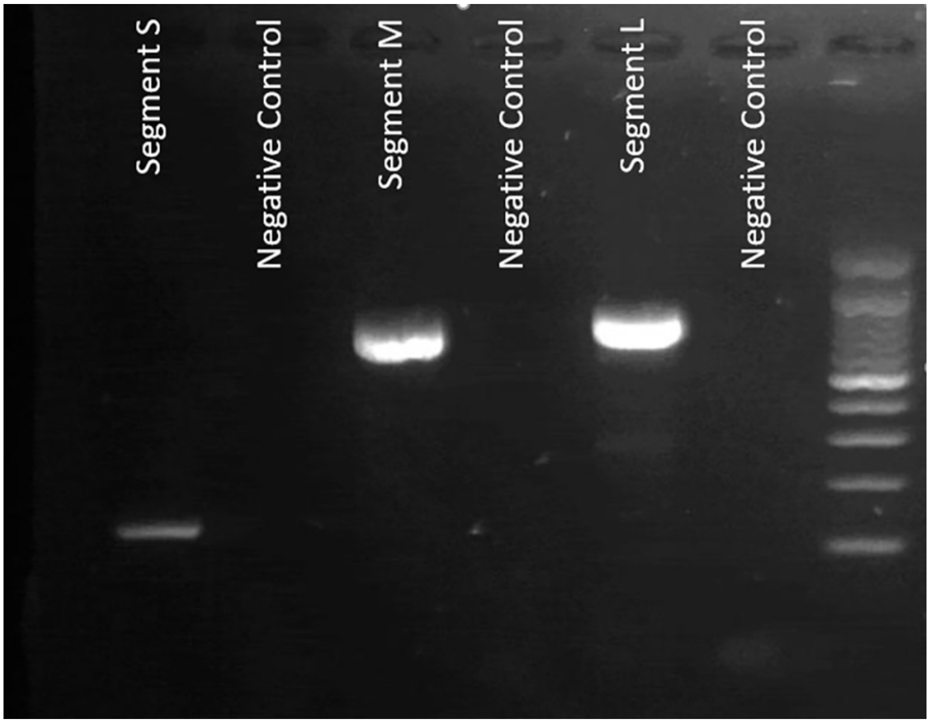

Appendix 1 Figure 1. Analysis of the large, medium, and small genomic segments of Cristoli virus from a 58-year-old woman, France A) Nucleotide (nt) sequence of the 3 genomic segments (L, M, and S) of Cristoli virus and their corresponding open reading frames (ORF). L, RNA-dependent RNA polymerase; Gn, envelope protein n; NSm, nonstructural protein m; Gc, envelope glycoprotein c; N, nucleocapsid; NSs; nonstructural protein s. B) Depth of sequencing per genomic position (i.e., exact number of times that a base in the reference sequence was covered by a read from sequencing experiments and aligned) for each segment of the Cristoli virus genome. C) Phylogenetic analyses of Cristoli virus genome segments L, M, and S with related members of the *Orthobunyavirus* genus of the *Peribunyaviridae* family. Bootstrap values are shown on the branches (ML model, 10,000 bootstraps). Scale bars indicate genetic variation per distance.

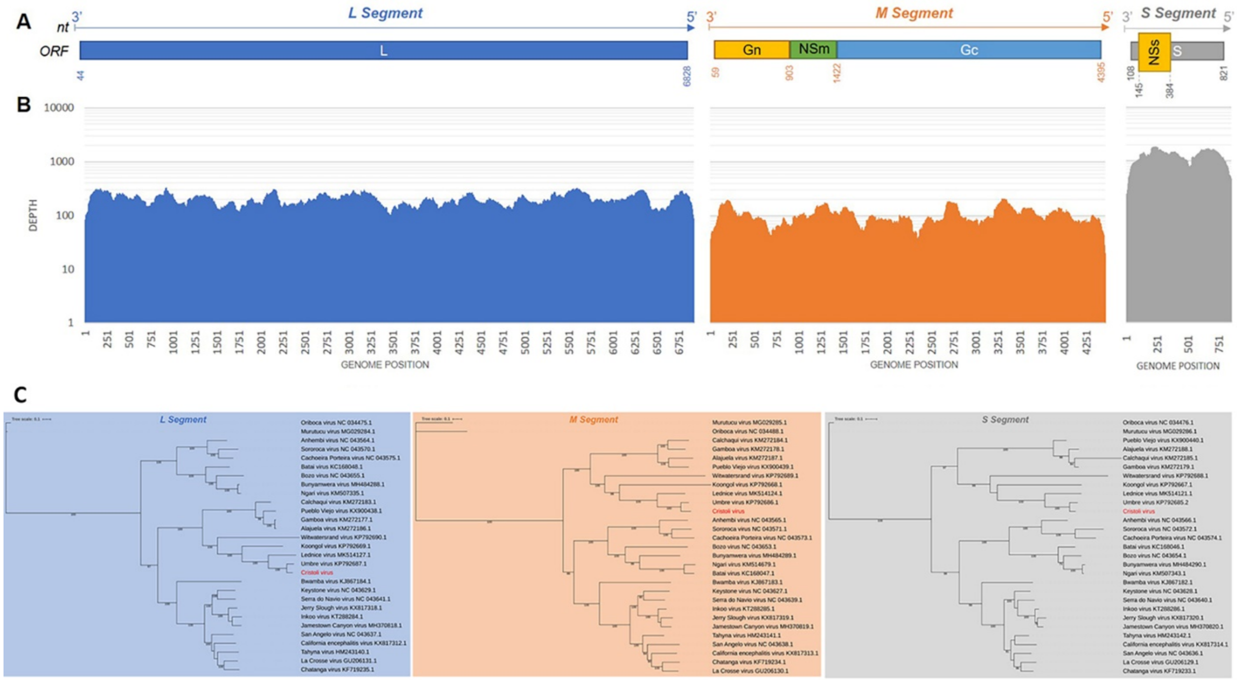

Appendix 1 Figure 2. Specific RT-PCR amplicons corresponding to Cristoli virus segments S (left, 150 bp), M (middle, 780 bp) and L (right, 857bp).
